# Minimizing Esophageal Heating During Radiofrequency Catheter Ablation

**DOI:** 10.1111/jce.70158

**Published:** 2025-10-26

**Authors:** Shayekh Abedin, Xingzhou Liu, Michael A. Barry, Stuart P. Thomas, Pierre C. Qian

**Affiliations:** ^1^ Department of Cardiology Westmead Hospital Sydney Australia; ^2^ Westmead Applied Research Centre, Faculty of Medicine and Health University of Sydney Sydney Australia

**Keywords:** atrial fibrillation, atrioesophageal fistula, catheter ablation, esophageal injury, esophageal temperature monitoring, metal proximity, pulmonary vein isolation

## Abstract

**Introduction:**

Atrio‐esophageal fistula is a rare but devastating complication from thermal esophageal injury during pulmonary vein isolation (PVI). Modifying ablation energy delivery and placement of esophageal temperature probes (ETPs) have been employed to mitigate the risk of thermal esophageal injury. Our study sought to compare the effects of return electrode location, insulation of the ETP, and use of high‐power short‐duration (HPSD) ablation on esophageal temperature, ETP temperature measurement, and thermal lesion dimensions.

**Methods:**

A myocardial phantom gel model using thermochromic material was used to approximate the atrio‐esophageal environment. Irrigated ablations were performed at 30 W/20 s (low‐power long duration [LPLD]) and 50 W/5 s (HPSD) with variation of return electrode position and insulation of the ETP. Temperatures were continuously logged, and digital photography with in‐house software was performed to examine lesion dimensions.

**Results:**

Distant placement of a return electrode compared to proximal placement decreased peak esophageal temperature (*T*_Peak_) across LPLD (41.73°C ± 0.12°C vs. 39.23°C ± 0.15°C; *p* < 0.0001) and HPSD ablations (39.17°C ± 0.06°C vs. 38.13°C ± 0.06°C; *p* < 0.0001). Insulation of the ETP reduced T_Peak_ across LPLD (39.13°C ± 0.25°C vs. 41.73°C ± 0.12°C; *p* < 0.0001) and HPSD ablation (39.17°C ± 0.06°C vs. 38.27°C ± 0.06°C; *p* < 0.0001). HPSD ablation produced lower mean *T*_Peak_ than LPLD regardless of the location of the return electrode location or insulation of the ETP.

**Conclusion:**

HPSD settings, insulation of the ETP, and a distant return electrode placement minimized esophageal heating. These strategies may reduce thermal injury to the esophagus during radiofrequency ablation.

## Graphical abstract

Esophageal temperature distribution profiles across the different experimental setups. The illustrations depict the temperature distribution across various ablation settings, utilizing thermochromic liquid crystal images from our phantom myocardial model. Each coronal image demonstrates radiofrequency ablation performed with the ThermoCool catheter on the gel surface, with the corresponding color gradient reflecting the specific ablation setting and circuit configuration. The dark blue color reflects the 53°C isotherm of necrosis, as can be seen on the color bar below, correlating to temperature in °C. A low‐power long‐duration (30 W for 20 s), near an insulated electrode with a local (posteriorly placed) return electrode.

**Abbreviations**
AF: atrial fibrillation
DRE: distant return electrode
EDEL: endoscopically detected esophageal lesions
ETP: esophageal temperature probe
HPSD: high‐power short‐duration
LET: luminal esophageal temperature
LPLD: low‐power long duration
LRE: local return electrode
PVI: pulmonary vein isolation
RF: radiofrequency
RFA: radiofrequency ablation
SD: standard deviation
TLC: thermochromic liquid crystal
T_Peak_
: peak esophageal temperature

## 1 Introduction

Radiofrequency catheter ablation (RFA) remains a common treatment for symptomatic, drug‐refractory atrial fibrillation (AF). Atrio‐esophageal fistula is a rare but devastating complication with a high mortality rate despite operative management [1]. Despite the development of strategies to detect and reduce the risk of thermal esophageal injury, subclinical injury with esophageal ulceration remains common where routine endoscopy is performed, occurring in up to 48% of patients [2, 3, 4].

Esophageal temperature probes (ETPs) have been introduced to monitor temperature in real time during ablation, thereby limiting excess heat exposure. However, its effectiveness in preventing esophageal injury is debated. A recent large, multicentre retrospective study reported a trend to reduced mortality from atrioesophageal fistula with ETP use [1]. In contrast to this, earlier studies suggest that ETP may cause mechanical injury or directly contribute to esophageal heating by facilitating radiofrequency (RF) current conductivity between ETP metal sensors and the ablation catheter [5, 6, 7]. Low‐power, long‐duration (LPLD) ablation at the thin posterior left atrial wall (PLA) is another common strategy used to limit intraluminal esophageal temperature rise and thermal damage [8]. However, the LPLD strategy results in greater amounts of conductive heating, which has the potential to create deeper lesions extending through the PLA into the esophagus, placing it at risk of thermal injury [9]. In contrast, high power, short‐duration (HPSD) is an alternative ablation strategy that creates shallower, broader ablation lesions through preferential resistive heating, limiting ablation depth and reducing the risk of thermal injury to the esophagus [10]. Finally, the location of the return electrode has been associated with differing ablation lesion depth as a function of current direction and circuit impedance [11, 12]. Variations in the anatomical location of the return electrode may therefore also influence esophageal heating.

The objective of this study was to examine the effect of ablation circuit configuration and settings, and insulation of ETP electrodes on esophageal heating. We hypothesized that: (1) HPSD compared to low‐power long‐duration (LPLD) ablation settings leads to less esophageal heating, (2) uninsulated electrode bearing ETPs can change the RF current path leading to passive electrode heating, and (3) RF current direction altered by return patch placement can influence esophageal heating.

## 2 Methods

### 2.1 Phantom Agar Model

**Figure 1:**
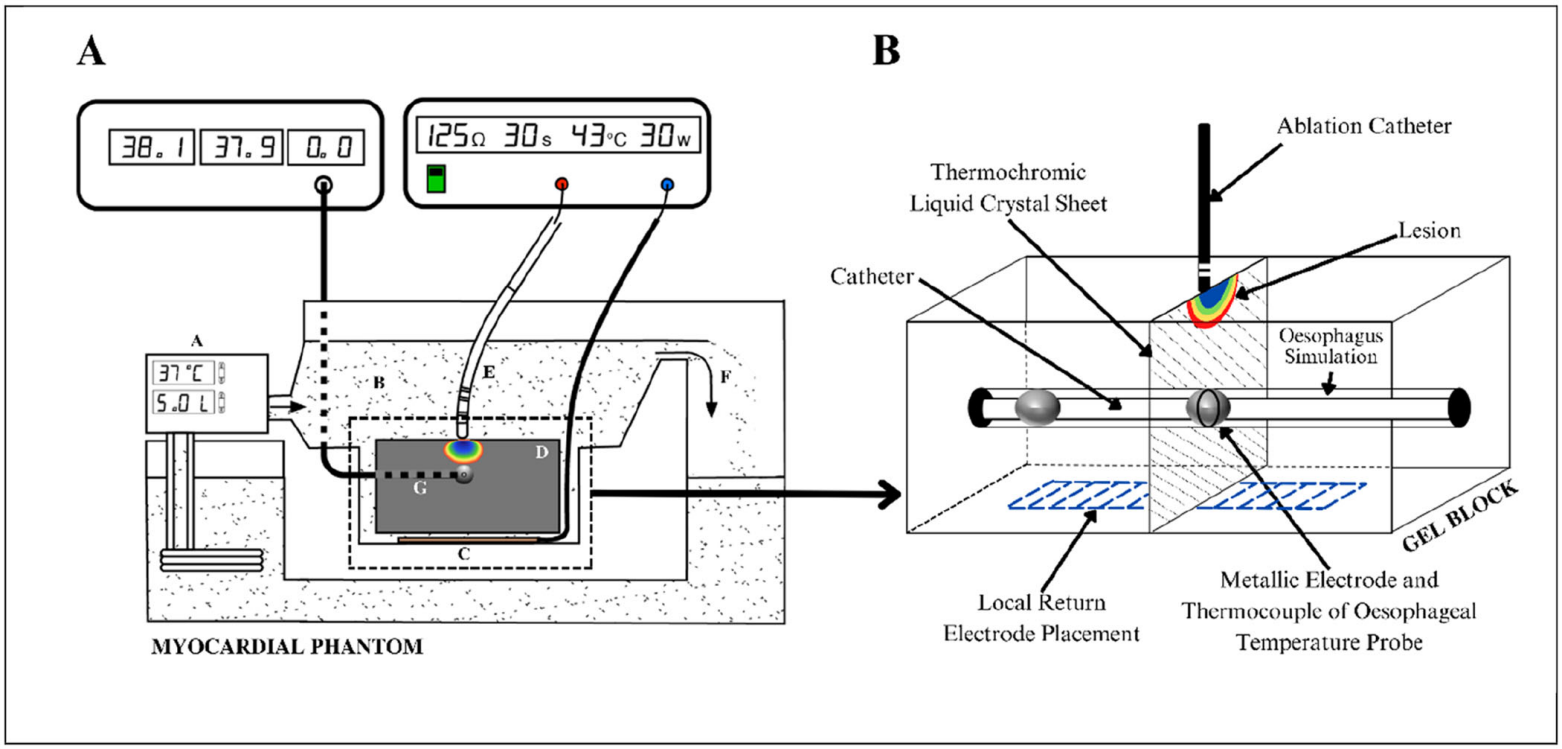
Myocardial phantom—Experimental preparation. (A) The phantom for RFA ablation, with the Perspex well setup containing SensiTherm within the esophageal lumen. A = pump and heater, B = supernatant fluid, C = return electrode location for distant return electrode/plate setup, D = perspex gel well with TLC, E = ThermoCool ablation catheter wired to its generating console, F = fluid drain to return to pump, and G = SensiTherm catheter wired to its console. (B) 3D Schematic diagram of radiofrequency ablation in the gel block of the phantom model. No air was present within the lumen as the gel was cast into it after the insertion of SensiTherm.

A myocardial phantom model [13] was constructed to mimic the atrio‐esophageal environment. A thermochromic liquid crystal (TLC) sheet was embedded in a gel composed of 1.5% w/v agar powder substitute (Phytagel, Sigma‐Aldrich, P8169) and 30% normal saline in deionized water (i.e., a 0.27% NaCl solution). A circular cut‐out was made to permit the transverse placement of an ETP (SensiTherm, Abbott Medical, Lake County, IL, USA) 4 mm from the gel surface, given that the posterior wall of the left atrium and the esophageal wall are each approximately 2 mm thick (Figure 1) [14, 15]. The TLC sheet displayed a color play corresponding to temperatures between 40°C and 58°C, which was specifically chosen to observe both sublethal (40°C–50°C) and lethal tissue hyperthermia ( > 53°C). To mimic blood flow in the left atrium, normal saline (0.9%) was heated to 37°C and flowed at a velocity of 55 mm/s, consistent with human endocardial environments [16]. Ethics approval was not required for this study as the experiments were conducted ex vivo using thermochromic gel and did not involve human participants or living animals.

### 2.2 Experimental Setup and Ablation Protocol

**Figure 2:**
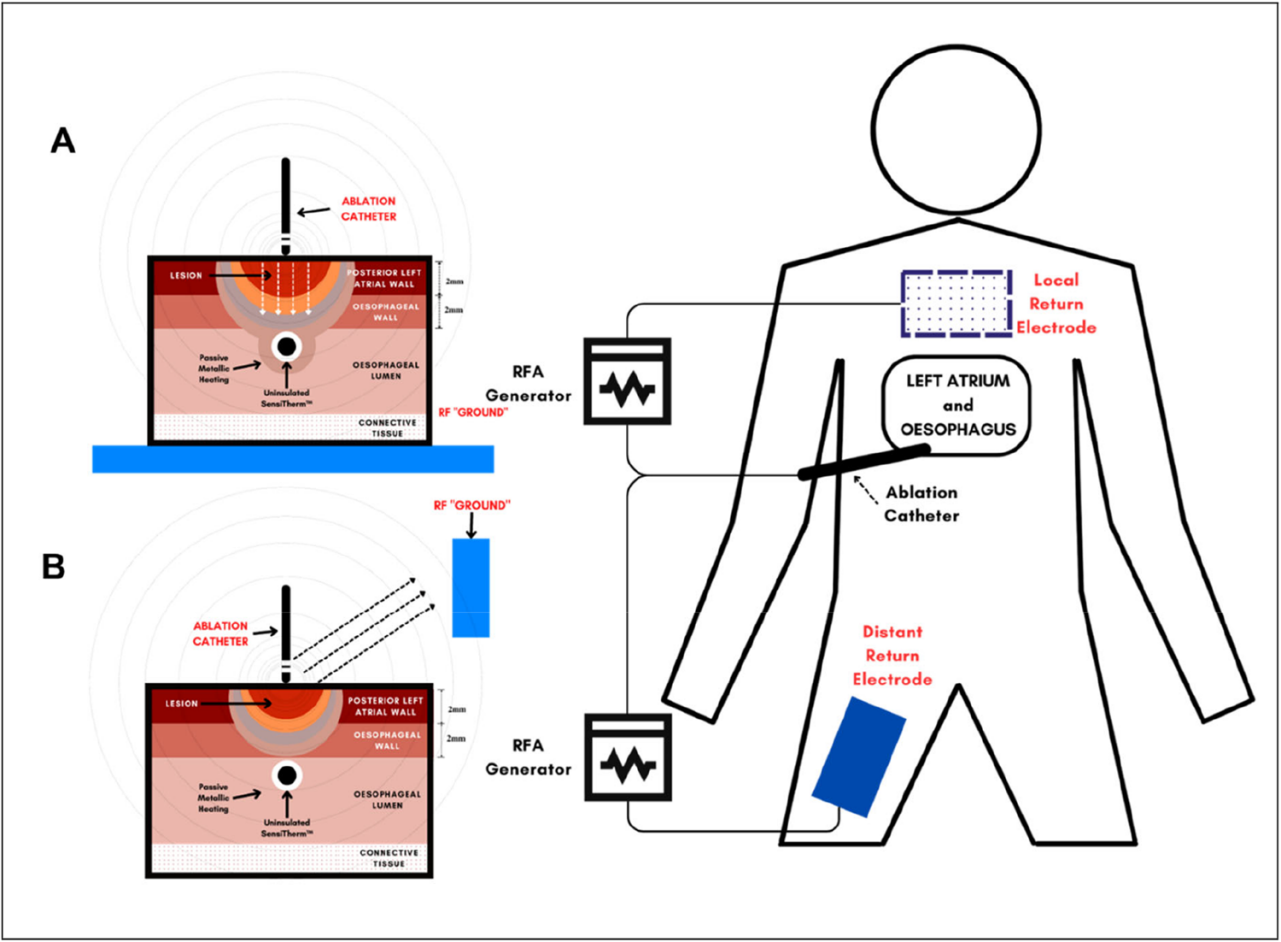
Schematic diagram illustrating the effects of return electrode positioning on esophageal heating during radiofrequency ablation (RFA), contrasting local (A) vs. distant (B) configurations across the gel model configuration (left), and the clinical setup (right).

An 8 F ablation catheter (Thermocool Surround Flow; Biosense Webster Inc., Diamond Bar, CA, USA) with a 3.5 mm irrigated tip electrode was positioned perpendicular to the TLC sheet, directly over the SensiTherm electrode. Contact force was not measured, but the model ensured uniform electrode surface contact through positional accuracy under direct visualization. Ablations were performed and delivered using the Stockert EP Shuttle Generator at two different settings: 30 W/20 s (LPLD) and 50 W/5 s (HPSD). An irrigation rate of 8 mL/min for LPLD and 15 mL/min for HPSD was employed, following the manufacturer's recommendations. Two ablation circuit configurations were tested to demonstrate the effects of dispersive electrode position on esophageal heating; in both, the baseline impedance was maintained at 100 Ω. In the first configuration, the return electrode was placed remotely, simulating a thigh position (Figure 2). The other configuration involved placing the return electrode such that the ETP was interposed between the ablation electrode and the return electrode, simulating a posterior chest location. Experiments were duplicated with both the SensiTherm metallic electrode exposed as well as electrically insulated with heat shrink to assess differences in lesion characteristics and electrode heating.

### 2.3 Measurement of Esophageal Probe, Tissue Temperatures, and Lesion Dimensions

The ETP temperature output was measured from the thermocouples within the SensiTherm electrodes, recorded through a high‐resolution digital camera focused on the SensiTherm console, and manually tabulated at 1 s intervals to generate esophageal temperature logs. Digital photography of ablation lesion formation was captured every 5 s using a Canon 5DmkII camera (Canon Inc., Japan) and a macro lens (USM EF 100 mm, Canon, Japan), providing a high resolution of approximately 90 pixels/mm. This setup also allowed for the observation of heat propagation in proximity to the SensiTherm metallic electrode. A hue‐temperature look‐up table was created for the TLC based on calibration against known temperature as previously described [13]. The isotherm of myocardial tissue necrosis was considered as 53°C. Lesion depth and width (mm) were measured using in‐house software (iChrome) in an automated fashion through isothermal mapping, with an accuracy of 0.5°C. Lesion volume was calculated using the half‐ellipsoid formula [17], v=(2×π×(w/2)²×d)/3, where *V* = volume (mm^3^), *w* = width (mm), *d* = depth (mm).

### 2.4 Statistical Analysis

Statistical analyses were conducted using GraphPad Prism 10.3.1 and R. Temperature data from SensiTherm were manually transcribed from video to GraphPad at 1‐s intervals and averaged across the three replicates. Descriptive statistics are presented as mean ± SD for continuous variables, unpaired Student's *t*‐test was used to compare between groups, with *p* < 0.05 considered statistically significant.

## 3 Results

### 3.1 HPSD Reduced Esophageal Heating, and Lesion Penetration Below Critical Depth, Compared With LPLD

HPSD resulted in lower peak esophageal temperatures (*T*_peak_), in both local and distant return electrode configurations (Tables 1 and 2). Ablation lesion depth was significantly reduced in the HPSD group compared to LPLD across local and distant return electrode configurations (Tables 1 and 2). HPSD resulted in significantly smaller lesion volumes than LPLD in the local return electrode configuration. In the distant electrode configuration, the ablation volumes produced by HPSD were comparatively smaller and did not reach statistical significance (Tables 1 and 2).

**Table 1:** Ablation lesion dimensions and esophageal heating with local return electrode configuration.

Local return electrode	LPLD	HPSD	*p*
Lesion volume (mm^3^)	75.25 ± 7.25	38.56 ± 2.07	0.0011
Lesion depth (mm)	3.74 ± 0.23	2.49 ± 0.04	0.0007
*T*_peak_ (°C)	41.73 ± 0.12	39.17 ± 0.06	< 0.0001

*Note:* HPSD resulted in smaller ablation lesion volume and depth with lower esophageal heating than LPLD in this configuration. Values are displayed as mean ± SD.

Abbreviations: HPSD, high‐power short‐duration; LPLD, low‐power long duration; T_peak_, peak temperature detected by esophageal temperature probe.

**Table 2:** Ablation lesion dimensions and esophageal heating with distant return electrode configuration.

Distant return electrode	LPLD	HPSD	*p*
Lesion volume (mm^3^)	19.32 ± 5.47	16.59 ± 1.40	0.4506
Lesion depth (mm)	2.32 ± 0.25	1.82 ± 0.06	0.0257
*T*_peak_ (°C)	39.23 ± 0.15	38.13 ± 0.06	0.0003

*Note:* HPSD resulted in decreased lesion depth and esophageal heating than LPLD in this configuration. Lesion volumes were comparatively smaller with HPSD compared to LPLD, but did not reach statistical significance. Values are displayed as mean ± SD.

Abbreviations: HPSD, high‐power short‐duration; LPLD, low‐power long‐duration; T_peak_, peak temperature detected by esophageal temperature probe.

### 3.2 Local Dispersive Electrode Placement Increased Esophageal Heating, and Lesions Sizes Compared to a Distant Configuration

**Figure 3:**
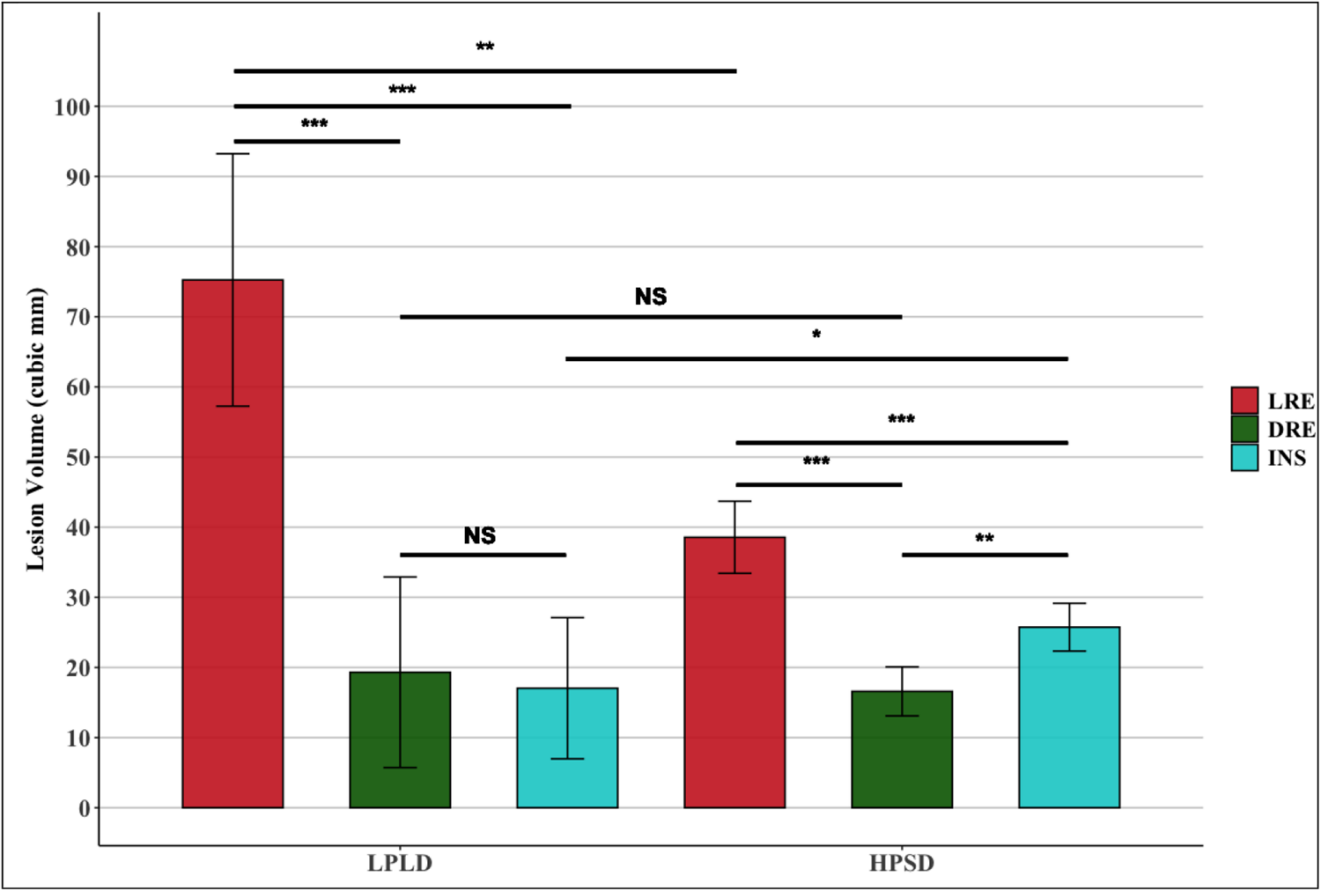
Ablation lesion volume with differing return patch placement and insulation of the esophageal probe at two different ablation settings. The local return electrode (LRE) configuration resulted in significantly larger lesion volumes compared to the distant return electrode (DRE) and insulated (INS) esophageal probe configurations. This relationship remained true across low‐power long‐duration (LPLD, 30 W/20 s) and high‐power short‐duration (HPSD, 50 W/5 s) ablation settings. Data are presented as mean with 95% confidence intervals. Statistical significance is denoted as follows *p < 0.05, **p < 0.01, and ***p < 0.001.

**Figure 4:**
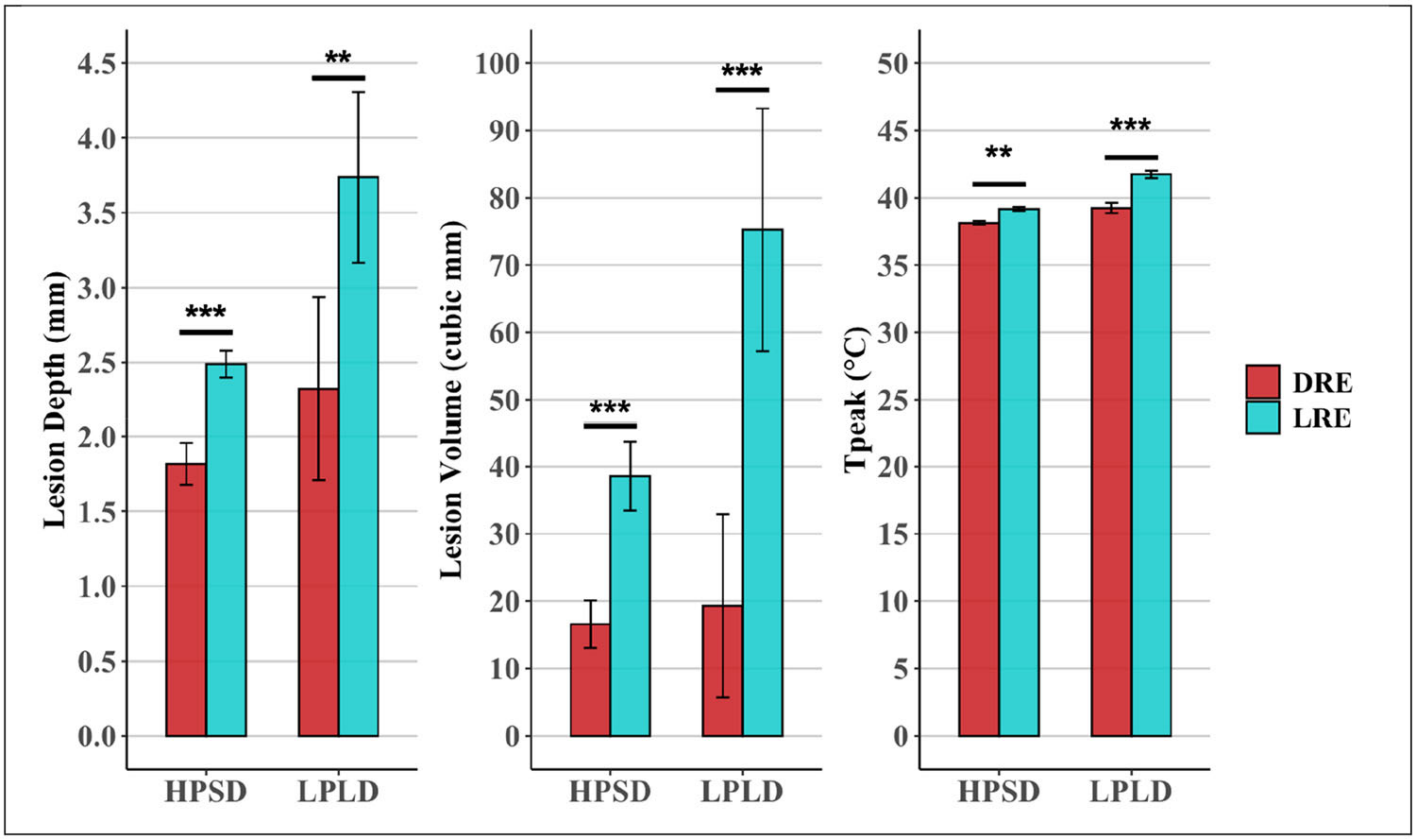
Ablation lesion dimensions and peak esophageal temperature readings with local and distant return patch placement. Ablation lesion dimensions were significantly increased with local return electrode placement (LRE) compared to distal return electrode placement (DRE) across both high‐power short‐duration (HPSD, 50 W/5 s) and low‐power long‐duration (LPLD, 30 W/20 s) ablations. The temperature peak (*T*_peak_) detected by the esophageal temperature probe during these ablations was significantly lower with DRE placement compared with LRE placement at these two ablation settings. Data are presented as mean with 95% confidence intervals. Statistical significance is denoted as follows *p < 0.05, **p < 0.01, and ***p < 0.001.

**Figure 5:**
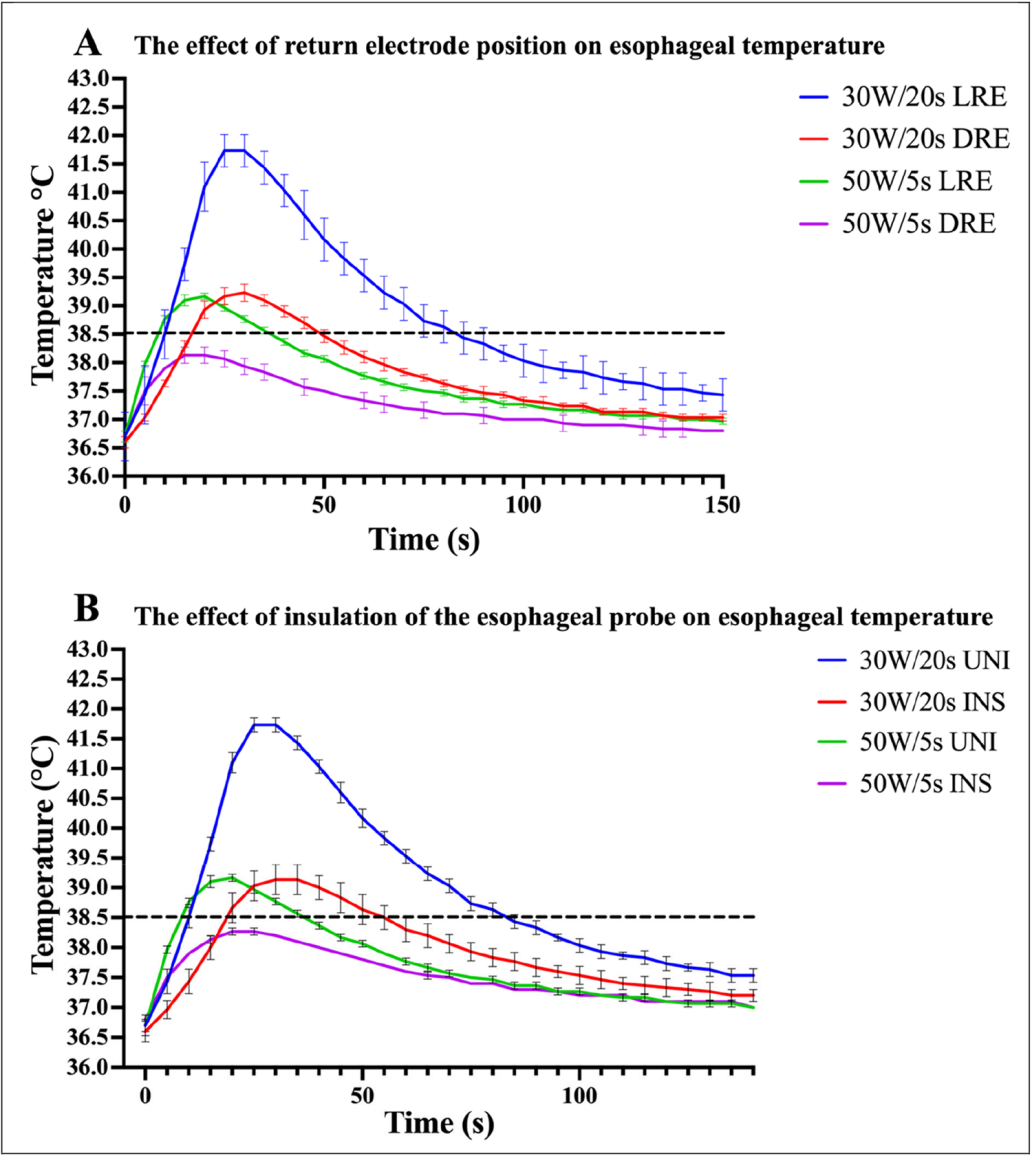
The effects of esophageal temperature probe insulation and return electrode placement on esophageal heating. The graph showcases the average temperature at the esophagus, measured through the esophageal probe, shown at each time point during ablation, with error bars reflecting the mean with 95% confidence intervals. The dashed line represents the temperature cutoff of 38.5°C. (A) The esophageal temperature profile compares the temperature kinetics of having a local return electrode position (LRE) compared to a distant return electrode position (DRE). (B) The graph shows the esophageal temperature profile with an insulated esophageal probe (INS) compared to an uninsulated esophageal probe (UNI) in the LRE configuration.

Ablation lesion dimensions and esophageal heating were influenced by the location of the local dispersive electrode (Figure 3). A local posteriorly placed dispersive electrode resulted in (1) increased lesion volume and depth (Figure 4), (2) higher *T*_peak_, and higher esophageal probe temperatures irrespective of whether LPLD or HPSD settings were chosen (Figures 4 and 5).

### 3.3 Esophageal Probe Insulation Reduced Esophageal Heating

**Figure 6:**
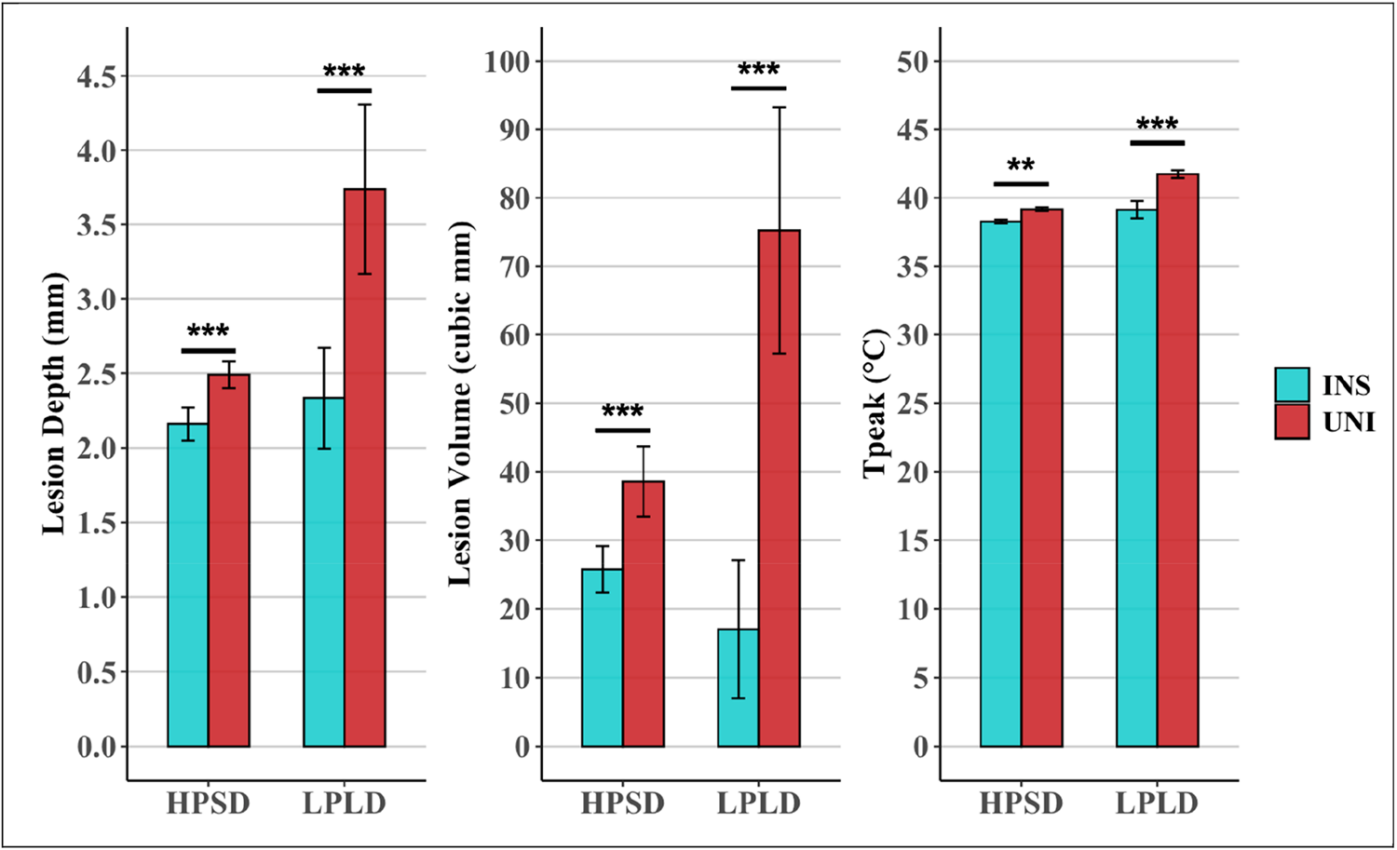
Impact of esophageal probe insulation on ablation lesion characteristics and esophageal temperature. The peak esophageal temperature was significantly lower when the esophageal probe was insulated (INS) across high‐power short‐duration (HPSD, 50 W/5 s) and low‐power long‐duration (LPLD, 30 W/20 s) ablations. The ablation lesion dimensions were significantly increased when an uninsulated probe (UNI) was utilized in this ablation setup across the above ablation settings. Data are presented as mean with 95% confidence intervals. Statistical significance is denoted as follows *p < 0.05, **p < 0.01, and ***p < 0.001.

Insulation of the esophageal probe reduced esophageal heating with a reduction in *T*_peak_ across LPLD and HPSD ablations (Figure 5). Both ablation lesion size and depth were significantly reduced with insulation of the esophageal probe across both power settings (Figure 6). Insulation of the ETP resulted in no observable remote metallic heating adjacent to the ETP according to the temperature distribution profile (Central Illustration 1).

**Central_Illustration 1:**
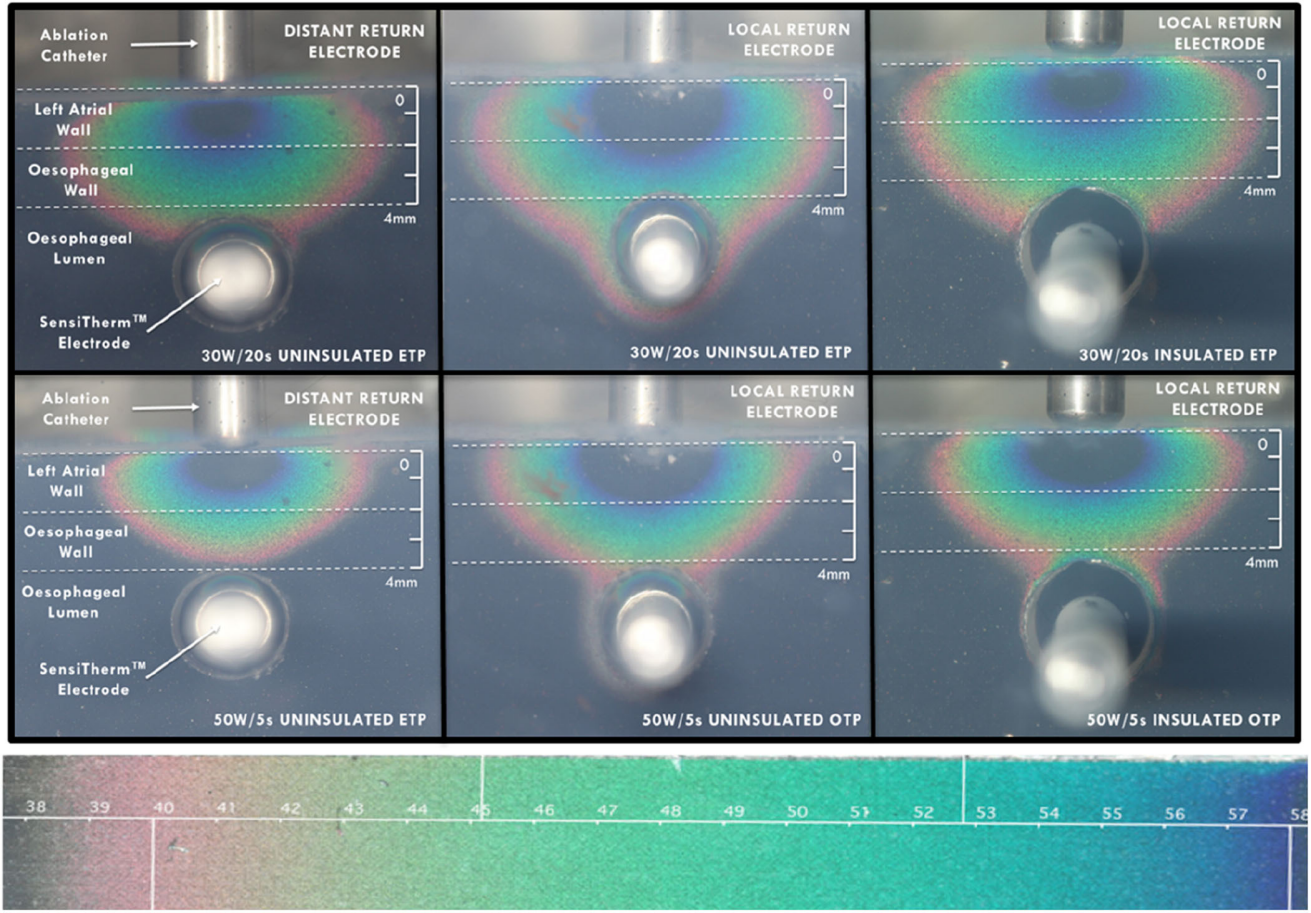
Esophageal temperature distribution profiles across the different experimental setups. The illustrations depict the temperature distribution across various ablation settings, utilizing thermochromic liquid crystal images from our phantom myocardial model. Each coronal image demonstrates RF ablation performed with the ThermoCool catheter on the gel surface, with the corresponding color gradient reflecting the specific ablation setting and circuit configuration. The dark blue color reflects the 53°C isotherm of necrosis, as can be seen on the color bar below, correlating to temperature in °C. A low‐power long‐duration (30 W for 20 s), near an insulated electrode with a local (posteriorly placed) return electrode.

## 4 Discussion

### 4.1 Study Results

In this study, we aimed to demonstrate basic biophysical principles related to radiofrequency ablation (RFA) at the posterior left atrium using an in vitro model. We showed that esophageal heating can be more pronounced during RFA with: (1) LPLD compared to HPSD settings, (2) a local posteriorly positioned return electrode compared to a distant return electrode, and (3) proximity to an uninsulated metallic ETP electrode compared to an insulated ETP (Figure 4).

### 4.2 HPSD vs. LPLD Ablation

A greater total energy was delivered with LPLD (30 W × 20s = 600 J) compared with HPSD (50 W × 5s = 250 J). The large quantity of heat energy produced enabled temperature rise at deeper strata from thermal conduction, leading to deeper lesions and higher esophageal temperature rises. By contrast, HPSD settings produce shallow ablation lesions where conductive heating is given less opportunity to deepen lesion dimensions due to the brevity of power delivery to generate a steep temperature profile and less esophageal heating. These findings are consistent with clinical studies showing that the incidence of endoscopically detected esophageal lesions with HPSD is not in excess of that reported with LPLD settings and is in the range of 2.0%–11.6% [18, 19].

### 4.3 Local vs. Distant Dispersive Electrode Placement on Esophageal Heating

Our data demonstrate that a local posteriorly positioned return electrode led to greater lesion dimensions and esophageal heating (Figures 3 and 4) in both LPLD and HPSD ablation settings. The configuration of return electrode placement has been found in previous animal and clinical studies to modulate RF lesion dimensions [12, 20, 21]. Specifically, placing the return electrode opposite to the catheter tip enhances current density into the intervening tissue due to the directionality of the RF current (Central Illustration 1). In contrast, if the return electrode is placed in an indifferent distant location, a larger portion of the current is dissipated into the blood pool due to the lower impedance of the blood compared with the myocardium, reducing tissue heating efficiency as shown in our model. The phenomenon of current directionality during RF ablation is most impressively demonstrated by bench models of bipolar ablation, which is an extreme example, as RF current is delivered across two closely positioned poles. In such a configuration, current directionality has such a strong influence on lesion creation that catheter‐tissue contact is not required for tissue heating [22]. Our center's clinical practice is to place the return electrode in a distal position; however, operators in different centers have reported adjusting the placement of the return patch on the chest to lower circuit impedance and direct increased current density, achieving larger ablation lesions and procedural success [12]. Our data suggest that the placement of the return electrode on the posterior chest during posterior LA ablation may enhance heating due to the additive nature of impedance reduction and altered current path directed through the esophagus. Therefore, consideration could be given to the placement of two distant dispersive electrodes to lower circuit impedance in patients with high body mass index if a lower circuit impedance is desired, rather than altering the position of the return electrode.

### 4.4 Esophageal Probe Insulation in Reducing Heating

Previous studies have linked the use of uninsulated ETPs with esophageal lesions [6]. Our findings demonstrate that uninsulated ETPS are not only associated with increased ablation lesion size but also contribute to remote heating around the metallic electrode present at both HPSD and LPLD ablation settings (Central Illustration 1). Uninsulated ETPs may mediate remote esophageal heating through resistive heating from altering the current path through the metallic electrode. This previously studied “antenna effect” could result in additional resistive heating of the surrounding luminal esophageal tissue [23]. By insulating the metallic electrode of the ETP, lesion dimensions were reduced and remote metallic heating was abolished at HPSD and LPLD settings tested, suggesting it is the presence of metal rather than the physical probe that led to this observed effect. This finding suggests that insulated ETPs should be used in preference to uninsulated ETPs to minimize the risk of thermal injury to the esophagus.

### 4.5 Strengths and Limitations

The in vitro model used has high spatial and temporal resolution, and is highly controlled to enable reproducible and consistent measurements to ascertain biophysical principles. Contact force was not measured due to the impressibility of the model, and instead, catheter positioning was visually guided. This highly controllable model enables a biophysical understanding of the factors that may influence esophageal heating, keeping all other variables the same; however, the effect size of these ablation setting configurations in preventing clinically significant esophageal injury cannot be obtained from this in vitro investigation, but are also otherwise difficult to obtain from clinical studies due to the low event of this serious complication.

## 5 Conclusion

We have shown that during RFA, esophageal heating may be minimized by choosing an HPSD setting, placing a distant return patch location, and using an insulated ETP. Understanding the biophysical principles demonstrated will help operators choose ablation settings for RFA with consideration of the local anatomical environment to achieve transmural ablation while avoiding collateral injury.

## Data Availability

The data that support the findings of this study are available from the corresponding author upon reasonable request.
